# Interactions among alcohol dependence, perinatal common mental disorders and violence in couples in rural Vietnam: a cross-sectional study using structural equation modeling

**DOI:** 10.1186/1471-244X-12-148

**Published:** 2012-09-19

**Authors:** Thach Duc Tran, Tuan Tran, Karen Wynter, Jane Fisher

**Affiliations:** 1 Research and Training Centre for Community Development, Hai Ba Trung District, 39/255 Vong street, Hanoi, Vietnam; 2 Jean Hailes Research Unit, School of Public Health and Preventive Medicine, Monash University, Clayton, Australia; 3 Melbourne School of Population Health, The University of Melbourne, Parkville, Australia

**Keywords:** Common mental disorders, Alcohol dependence, Domestic violence, Perinatal period, Couples, Vietnam

## Abstract

**Background:**

There is increasing recognition that perinatal common mental disorders (PCMDs) are prevalent in women in low and lower-middle income countries and emerging evidence that PCMDs and alcohol abuse occur in men in these settings. Domestic violence is associated with PCMDs in both women and men. The aim of this study was to examine the relationships among PCMDs, alcohol abuse and domestic violence in couples in a rural, low-income setting.

**Methods:**

A cross-sectional, population-based study was undertaken in randomly selected communes in Ha Nam and Hanoi, Vietnam. All women in the selected study sites who were at least 28 weeks pregnant or were mothers of 4 – 6 week old babies in the recruitment period were eligible. The husbands of the women who consented to join the study were also invited to participate. Data sources were study-specific questions and standardised measures: PCMDs were assessed by psychiatrist-administered Structured Clinical Interviews for DSM IV disorders, and alcohol dependence (AD) by the CAGE questionnaire (cut-off of ≥ 2). Structural Equation Modeling was used to test direct, indirect and mutual relationships simultaneously in the hypothesised model.

**Results:**

In total 364/392 (93%) eligible women agreed to participate. Of these, 360 were married, and 230 (64%) of their husbands also participated to yield a sample of 230 couples for analyses. Overall, in 7.4% (95% CI: 4.6-11.6) of couples both wife and husband were diagnosed with a PCMD; and 41.2% (95% CI: 35.1-47.8) of couples at least one member had a PCMD. Comorbid PCMD and AD were observed in 6.9% (95% CI: 4.3-11.0) of men, but did not occur in women. After controlling for other psychosocial risk factors comorbid PCMD and AD in husbands increased by 4.7 times the probability of PCMDS in their wives via intimate partner violence. PCMDS in wives did not increase the probability of PCMDS or AD in husbands.

**Conclusions:**

These data provide evidence that comorbid PCMD and AD in husbands have a significant adverse effect on the mental health of their wives in rural areas of Vietnam. This indicates that strategies to prevent and treat PCMDs in women will be more effective if paired with initiatives to reduce alcohol dependence and violent behaviours in men.

## Background

Perinatal common mental disorders (PCMDs) are non-psychotic mental health conditions including depressive, anxiety, adjustment and somatoform disorders which occur during pregnancy or in the postpartum year, compromise day-to-day functioning and are identifiable in primary health care [1]. There is increasing recognition that PCMDs are more prevalent among women living in low and lower-middle income than in high-income countries and emerging evidence that they also occur, albeit at lower rates, among men [2-4]. Both maternal and paternal PCMDs exert adverse effects on child outcomes [5,6]. However, the relationships between paternal and maternal PCMDS in couples remain unclear.

There are a number of reports of paternal PCMDs occurring in conjunction with and / or in response to maternal PCMDs [7-11]. The correlation coefficients of depression in one partner with depression in the other vary from 0.25 in the United States [7] to 0.75 in Poland [8]. A recent meta-analysis of 43 studies estimated a moderate pooled correlation coefficient of 0.31 [3]. Several studies have attempted to examine the relationship after controlling for socio-demographic characteristics and have concluded that women’s PCMDs adversely affects their partners’ mental health status [8-11]. However, a significant correlation does not indicate a causal relationship. Because they share living conditions, co-occurring disorders in women and men might be attributable to the same risk factors. It is also plausible that the relationship might operate in the other direction: that CMD in men contribute to PCMDs in their partners. Overall, there is as yet, little clear evidence for the direction of the relationship between PCMDs in women and men [3].

Alcohol-related problems can accompany common mental disorders [12]. Several explanations are offered for this co-occurrence, including increased risk of vulnerability for a second disorder as a result of the primary disorder, and common underlying genetic and environmental risks [12,13]. A systematic review of thirty-five studies conducted in both developing and developed countries reported a pooled prevalence of 16% (range 6-57%) of current alcohol problems and 30% (range 10-60%) of lifetime alcohol problems in people with depression [14]. In Vietnam, this comorbidity is rare in women, but we found that the current prevalence of AD was 6.9% in men and 38.9% in men who had a perinatal common mental disorder [15]. Comorbidity leads to a higher risk of suicide, and greater social and personal impairment, especially violent behaviour [16]. Nevertheless, the effect of this comorbidity on the mental health status of the partner has not been studied. The aim of this study was to examine the relationships between PCMDs in wives and comorbid PCMDs and alcohol dependence in husbands in Vietnam. This study used Structural Equation Modeling to examine direct and indirect relationships amongst variables of interest simultaneously. This cannot be achieved by conventional multiple linear or logistic regression analyses because those techniques are only able to take one dependent variable at a time into account.

## Methods

We report data from a community-based cross-sectional study conducted in randomly selected study sites in Ha Nam, which is a typical Red River delta rural province, and Hanoi, the national capital of Vietnam and a major urban centre.

### Participants

The sample size of this study was calculated to estimate the prevalence of PCMD in women and their husbands. The required sample size was equal to or more than 200 couples with the assumptions that prevalence of PCMD was 30% in women and 15% in men and a precision of 6.5%. This sample size allows including at least 20 variables into a statistical model [17].

A two-stage sampling protocol was used to generate a random selection of 6 communes in Ha Nam and 4 communes in Hanoi. All of the women in the selected communes who were at least 28 weeks pregnant or were mothers of 4 – 6 week old babies in the recruitment period and the husbands of the women who consented were eligible and invited to participate [18-20]. We use the terms “husband” and “wife” to describe the men and women who participated in the study as all couples are married and this is the preferred descriptor in this setting.

### Materials

Data sources were standardised instruments and a study-specific questionnaire.

*Perinatal common mental disorders*: All participants completed individual psychiatrist-administered Structured Clinical Interviews for the DSM IV Axis 1 Diagnoses (SCID-I) modules for depression, generalised anxiety, and panic disorder [21]. This is the demonstrated gold standard for diagnosis of perinatal mood disorders in diverse cultural settings and countries [22].

*Alcohol dependence (AD)* was assessed using the CAGE questionnaire [23]. This is a brief and widely used screening instrument which includes four questions with yes / no response options: Have you (1) felt the need to ***C****ut* down your drinking, (2) felt ***A****nnoyed* by criticism of your drinking, (3) had ***G****uilty* feelings about drinking, and (4) taken a morning ***E****ye* opener. The CAGE has been validated in many countries [24]. Most of the validation studies including those in low-income settings have found that agreement with at least two of the four items detects alcohol abuse or dependence with more than 80% sensitivity and specificity [24].

*Experience of intimate partner violence* was measured by experiences in three domains: fear of and actual physical violence, controlling behaviours, and absence of affection and care. Experiences of fear of and physical abuse were assessed by two study-specific questions: “*In the past year, have you ever felt frightened of your partner?*” and “*Within the last year have you been hit, slapped, pushed, kicked or otherwise physically hurt by your partner?”* We used the 24-item Intimate Bond Measure [25] – Vietnam to assess quality of intimate partner relationship It yields two subscales, Control and Care. The "Care" factor reflected perceived sensitivity, warmth, emotional responsiveness, trust, physical and capacity for companionship. The other dimension, labelled “Control” reflected perceived coerciveness, exertion of power or dominance and extent of criticism. Scores on each subscale range from 0 to 36, with higher scores on the Control subscale indicating less optimal and on the Care subscale more optimal behaviours towards the intimate partner. We have shown that this Intimate Bond Measure – Vietnam is meaningful and comprehensible to Vietnamese men and women [26].

*Socio-demographic characteristics* that were assessed included age and marital, educational, and occupational status. Information about 17 household characteristics, services and durable assets was collected to calculate a household wealth index following the World Bank method [27]. Current coincidental life adversity was assessed by an open-ended question: “*Apart from being pregnant* [or having recently had a baby] *are there other events in your life now that are worrying or distressing?*” Those who answered in the affirmative were asked an open-ended follow up question seeking a brief description of the source of worry. The quality of relationship with mother and mother-in-law was assessed by means of single fixed-choice items assessing trust and affection in each of these relationships. A question of similar format was used to assess whether participants were frightened of other family members, and if yes, a follow up question to ask who they were afraid of.

### Procedure

In Vietnam self-report questionnaire completion is unfamiliar and data were therefore collected in individual structured interviews which are preferred [28]. Husbands and wives were interviewed privately and separately at commune health stations by a member of the Research and Training Centre for Community Development (RTCCD) health research team. A senior Vietnamese psychiatrist from RTCCD administered the SCID-Is in separate individual interviews. Data collection was carried out in Ha Nam in November 2006 and in Hanoi in February and March 2007. All assessments were completed by both wives and husbands.

### Analysis strategy

Data management and univariate statistical analyses were performed in STATA version 11 (StataCorp LP, College Station, Texas, United States of America, 2009). Any person diagnosed with a mild, moderate or severe major depressive episode (DSM-IV codes 296.21, 296.22 and 296.23), a dysthymic disorder (DSM-IV code 300.4), panic disorder with or without agoraphobia (DSM-IV codes 300.01 and 300.21) or generalized anxiety disorder (DSM-IV code 300.02) was classified as having a PCMD. People who endorsed at least two of the four items of CAGE AD were classified as having AD. Socioeconomic position was assessed by a household wealth index which is a well established “proxy” measure [27]. Participants in the lowest quintile of the household wealth index were classified as being in a low socioeconomic position. An intimate partner violence index was constructed by confirmatory factor analysis of the items contributing to the IBM Care score, IBM Control score and any experience of fear of / or physical abuse from the marital partner. A higher score on the intimate partner violence index indicates that more violence has been experienced. Two variables, husband’s PCMD and alcohol dependence, were combined into one variable with four categories: neither PCMD nor AD; only PCMD; only AD; and comorbid PCMD and AD. Data were constructed in a couple structure in which each record was the data of a couple. Only data from couples in which there was no missing information from either partner were included.

Structural Equation Modeling analysis (SEM) was performed in Mplus version 6 (Muthén & Muthén, Los Angeles, United States of America (1998–2011)) to examine the relationships among variables. Two models were tested. In the first model, the indirect effect of comorbid PCMD and AD in husbands on PCMDs in wives was tested with intimate partner violence as a mediator, controlling for other potential psychosocial risk factors. The second model tested the same relationships with PCMD in husbands as the outcome.

The main outcomes, having or not having a PCMD in the wife or the husband, were binary variables, therefore a weighted least square estimator (WLSMV) was used to estimate unknown parameters with a probit link function. For continuous outcomes (e.g. intimate partner violence index), SEM produces linear regression coefficients which can be interpreted as one coefficient unit increase in the outcome for every unit increase in the predictor, if other predictors are kept constant. For binary outcomes (e.g. PCMD), probit regression coefficients are produced. Probit regression coefficients represent the change in the probit index for each unit change in the predictor. Indirect and direct effects were calculated, and tested for statistical significance. The probit coefficients were converted into probabilities by the formula (1) recommended by Muthen B.O [29].

(1) $$\mathrm{Probability}\;\left(y=1|x\right)\;=\;\Phi \left(-\beta_{0}+\beta_{1}x\right)$$

where y is the binary outcome, x is the exposure, Φ is the standard normal distribution function, β_0_ is the threshold, and β_1_ is estimated probit coefficient of x.

In order to evaluate model fit, we used Chi-Square Test of Model Fit with p values greater than 0.05 indicating a good fit, Root Mean Square Error Of Approximation (RMSEA) with values less than 0.05 indicating a good fit, and Tucker-Lewis Index (TLI) and Comparative Fit Index (CFI) with values greater than 0·95 indicating a good fit [30].

### Ethics

The study was approved by the University of Melbourne’s Human Research Ethics Committee and the Vietnam Medical Association’s Scientific Committee.

## Results

### Sample characteristics

In total 364/392 (93%) eligible women agreed to participate, of whom 360 were married. Overall, 230/360 (64%) of their husbands also participated. The most common reasons for non-participation were that men were not currently living in the rural province because they were working on construction projects in Hanoi or their work commitments precluded attendance at the commune health station in business hours [20]. There were no significant differences in the sociodemographic characteristics or the prevalence of PCMDs in women whose husbands did or did not participate. Only the 230 couples in which both wife and husband participated were included in the analyses reported in this paper.

The mean age of the 230 women was 26.6 years (SD = 5.4, range 17 to 43 years old). A substantial proportion (17.4%) of the women had not completed secondary school and 75.6% generated income through agricultural or manual work. The husbands ranged in age from 20 to 49 years and were on average 31 (SD = 6.3) years old. A similar proportion of them had not completed secondary school as their wives (16.5%), and 82.2% generated income through agricultural or manual work. More detailed characteristics of the sample can be found in previous papers [18-20].

### Perinatal common mental disorders

The prevalence of PCMDs in individuals and couples is shown in Table 1. The prevalence of PCMD in wives was significantly higher than in husbands (p < 0.001). Alcohol dependence was prevalent in husbands but not reported at all in wives.

**Table 1:** Prevalence of perinatal common mental disorders in 230 couples in Vietnam

	**Number (N=230)**	**Percentage (95% CI)**
**Wives**		
PCMD	71	30.9 (24.8-36.9)
Alcohol dependence	0	0
**Husbands**		
PCMD only	25	10.8 (7.4-15.6)
Alcohol dependence only	62	26.8 (21.6-33.1)
Comorbid PCMD and Alcohol dependence	16	6.9 (4.3-11.0)
**Couples**		
Wife PCMD only	54	23.4 (18.4-29.4)
Husband PCMD only	24	10.4 (7.1-15.1)
Both wife and husband PCMD	17	7.4 (4.6-11.6)
At least one with a PCMD	95	41.2 (35.1-47.8)

### Relationship between comorbid PCMD and AD in husband and wife’s PCMDs

**Figure 1:**
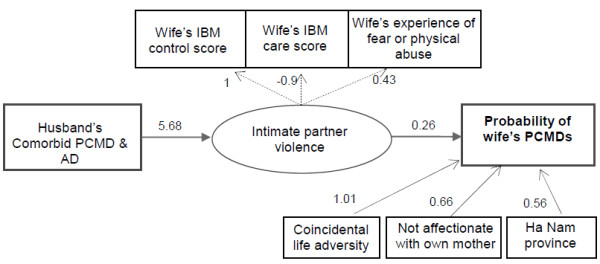
**Indirect effect of comorbid PCMD and AD in husbands on probability of PCMD in wives via intimate partner violence.** Only significant paths were included (see Table 2 for details). All of the variables in the diagram (presented in rectangular boxes) are observed except for the unmeasured (latent) variable ‘Intimate partner violence’ (represented as an ellipse).Single-headed solid arrows represent statistically significant directional paths, whereas dashed lines indicate factor loadings of the latent variable.

Structural Equation Modeling was used to test simultaneously the direct and indirect paths among husband’s PCMD and alcohol dependence, wife’s PCMDs, possible mediators and background variables. The main significant paths are shown in Figure 1, while the full model is presented in Table 2. The fit indices indicate that the model fits the data very well, in that all of the indices were within the range of perfect fit.

**Table 2:** Full structural equation model (SEM) to predict perinatal common mental disorders in 230 women in Vietnam

**Parameter estimates**	**SEM coefficient**	**Standard Error**	**p-value**
*Wife’s PCMDs*			
Intimate partner violence	0.26	0.12	0.02
Husband PCMD and AD			
Neither PCMD nor AD	*(reference)*		
Comorbid PCMD and AD	5.5	4.5	0.22
Only PCMD	0.04	0.3	0.89
Only AD	-0.10	132	0.99
Province: Ha Nam	0.56	0.2	0.03
(Wife) Affectionate and trusting relationship with own mother: No	0.66	0.2	0.008
(Wife) Coincidental life adversity: Yes	1.01	0.3	<0.001
(Wife) Fear of other family members: Yes	0.3	0.4	0.47
*Intimate partner violence*			
Husband PCMD and AD			
Neither PCMD nor AD	*(reference)*		
Comorbid PCMD and AD	5.68	1.3	<0.001
Only PCMD	0.37	0.6	0.58
Only AD	0.28	167	0.99
(Husband) age (in years)	-0.07	0.04	0.11
(Husband) education: Year 9 and less	0.09	0.68	0.89
(Husband) permanent income: Yes	0.33	0.58	0.57
(Wife) Affectionate relationship with mother-in-law: No	0.9	0.55	0.10
First child: Yes	1.04	0.8	0.19
Household wealth index: Low	0.72	0.7	0.36
*Intimate partner violence defined by*			
Wife’s perception of control from her husband (wife’s IBM Control score)	1.00		
Care from husband to wife (wife’s IBM Care score)	-0.9	0.28	0.001
Fear of or experience of physical abuse from the husband	0.43	0.12	<0.001
*Effect from husband comorbid PCMD and AD to probability of wife’s PCMDs*			
Indirect effect via intimate partner violence	1.50		0.02
Direct effect	5.54		0.23
*Thresholds*	**Estimate**		
Wife’s PCMD	1.07		
Intimate partner violence	1.70		
Fit indices	**Estimates**		
χ^*2*^/*df* (p-value)	41.5/49 (p=0.767)		
RMSEA (Probability RMSEA <= .05)	<0.001 (p=0.99)		
CFI	1.00		
TLI	1.00		

The probability of wife’s PCMDs was increased significantly by comorbid PCMD and AD in the husband via an indirect path, intimate partner violence, but this relationship was not observed in couples where the husband had only alcohol dependence or only PCMD. The direct path, however, was not significant. The probability of the wife having any PCMD if the husband had no comorbid PCMD and AD was 0.14, while if the husband had comorbid PCMD and AD this increased 4.7 times to 0.66.

The regression of intimate partner violence shows that only comorbid PCMD and AD was a risk factor, not other characteristics in the woman or her husband. A positive coefficient (5.68, p < 0.001) shows that women married to men with co-morbid PCMD and AD were more likely to perceive the relationship as being of poorer quality as indicated by the intimate partner violence index.

SEM results confirmed the results of a multiple logistic regression of wife’s PCMDs reported in previous publications [18]. Living in rural areas, poorer relationship with own mother and coincidental life adversity along with poor marital relationship were the determinants of women’s PCMDs.

### Relationship between PCMDs in wife and husband’s PCMDs

A similar model was tested which examined the direct and indirect pathways from the wife’s PCMDs to the husband’s PCMDs and AD (Table 3). The coefficient of the direct pathway was 0.508 (p = 0.14), while the indirect pathway via intimate partner violence towards the husband was −0.113 (p = 0.70). These results suggest that there is no significant effect of PCMDs in the wife on husband’s PCMDs and/or AD.

**Table 3:** Full structural equation model (SEM) to predict perinatal common mental disorders in 230 men in Vietnam

**Parameter estimates**	**SEM coefficient**	**Standard Error**	**p-value**
*Husband’s PCMDs*			
Intimate partner violence	0.57	0.24	0.01
Wife’s PCMD	0.51	0.34	0.13
Province: Ha Nam	-0.5	0.53	0.34
(Wife) Affectionate and trusting relationship with own mother: No	0.20	0.28	0.49
(Wife) Coincidental life adversity: Yes	0.62	0.23	0.001
(Wife) Fear of other family members: Yes	1.00	0.67	0.13
*Intimate partner violence*			
Wife PCMD	-0.19	0.50	0.69
(Wife) age (in years)	0.03	0.07	0.62
(Wife) education: Year 9 and less	0.09	0.51	0.85
(Wife) permanent income: Yes	-0.04	0.50	0.92
(Husband) Affectionate relationship with mother-in-law: No	0.97	0.46	0.03
First child: Yes	-0.14	0.71	0.83
Household wealth index: Low	-0.12	0.17	0.49
*Intimate partner violence defined by*			
Husband’s perception of control from his wife (Husband’s IBM Control score)	1.00		
Care from wife to husband (Husband’s IBM Care score)	-0.63	0.33	0.06
Fear of or experience of physical abuse from his wife	0.59	0.24	0.01
*Effect from wife PCMDs to probability of husband’s PCMDs*			
Indirect effect via intimate partner violence	0.39	0.27	0.14
Direct effect	-0.11	0.29	0.69
*Thresholds*	**Estimate**		
Husband’s PCMD	1.54		
Intimate partner violence	0.55		
Fit indices	**Estimates**		
χ^*2*^/*df* (p-value)	47.1/70 (p=0.93)		
RMSEA (Probability RMSEA <= .05)	<0.003 (p=0.98)		
CFI	0.99		
TLI	0.99		

## Discussion

This study has considerable strengths, which distinguish it from prior research about PCMDs in women and men in couples. We recruited a representative sample of couples systematically from a community population and used a gold standard psychiatrist-administered structured clinical interview to provide reliable diagnoses of PCMDs. The relationships between husband’s PCMDs and wife’s PCMDs during the perinatal period were examined in both directions using the same Structural Equation Modeling procedure. We found that comorbid PCMD and AD in husbands increased the probability of PCMDs in wives through the perpetration of violence. The magnitude of the effect was statistically and clinically significant (an increase of 4.7 times, p = 0.02). In the opposite direction, PCMDs in wives did not affect the probability of PCMDs in husbands. Perinatal mental health problems in men have been somewhat neglected because they may not be as prevalent as in women [3], but the body of evidence is increasing. Our findings suggest that the conclusions of several investigators that husbands of women with PCMDs are at increased risk of PCMDs, are not necessarily accurate and rather that the relationship might be in the other direction, [8-11,31]. There are several potential explanations for the differences.

First, some previous studies [7-11] have used multiple regression or logistic regression procedures to examine the relationship between husband’s PCMDs and wife’s PCMDs. These techniques violate the assumption of independence of data, if data come from members of the same couples and do not allow indirect relationships to be detected. However, this limitations are overcome by using the more advanced Structural Equation Modeling [30] technique. In this study, we used SEM to investigate whether there was an indirect effect via domestic violence, which is usually either not assessed or treated as a confounder.

Second, the studies that have investigated the relationship between PCMDs in women and the risk of PCMDs in men have been conducted in high-income countries. While globalisation is contributing to changes in traditional gender roles, which have been the norm in countries like Vietnam, these changes are occurring in urban, and not yet in rural farming communities. Men still occupy a dominant position in these families in which they do not usually share household tasks or infant care and in which they retain control over financial expenditure. Women have limited autonomy and less access to education and income-generating work than men do. Although domestic violence is a crime in Vietnam there is little social protection and as yet few services for women seeking to leave abusive relationships [32]. Women are more confined to their households in advanced pregnancy and the early postpartum period and it is plausible that domestic violence is especially psychologically harmful to them at this life stage when there are few avenues of escape and they are seeking to protect the fetus or newborn. Gender inequality increases risk of gender-based violence and these data contribute further evidence of the adverse impact of intimate partner violence on women’s mental health [33] in the perinatal period.

Finally, prior studies did not assess alcohol dependence. AD is prevalent in men in many societies and is linked to the perpetration of domestic violence [34,35]. The prevalence of comorbid psychiatric and alcohol use disorders is well established in general populations in many countries [14]. However, the effects of this comorbidity on the mental health of their intimate partners during the perinatal period, have not been examined. Our data revealed that if husbands in this setting had only a PCMD or only an AD then it did not affect their partners’ perinatal mental health status. The clinically significant pathway was between comorbid PCMD and AD in husbands to PCMDs in wives via the perpetration of gender-based violence.

These data provide crucial community-based evidence to shape policy for the prevention and management of perinatal common mental disorders in low-income countries. First, they indicate that both women and men should be included, and that there should be a targeted focus on couples in which the husband has both PCMD and AD. Second, alcohol dependence and intimate partner violence should be addressed in attempts to improve mental health care for men and, indirectly, for women. Finally they suggest that community-based gender education has an important role in the promotion of perinatal mental health in resource-constrained settings.

As data are collected simultaneously in studies with cross-sectional designs, the direction of relationships between variables cannot be attributed. Over last two decades, however, SEM analysis techniques have been developed to allow the testing of hypothesised directional relationships in cross-sectional studies [36]. In this study, the structural equation model fitted the data very well, which permits us to make conclusions about the direction of relationships between risks and outcomes with confidence.

We acknowledge the limitation that we were unable to invite and therefore could not recruit a high proportion of husbands (64%) and this might have had an effect on the findings in either direction. However, as there were no significant differences between the socio-demographic characteristics and mental health status of the wives whose partners did or did not participate we believe that any effects would be minimal and unlikely to affect the conclusions. Another limitation was that one measure used in this study the CAGE questionnaire was not validated for local use. As is often the case in resource-constrained settings, validated tools were not always available. The tools we selected have been used and validated in Vietnam and provided interpretable data in previous studies.

## Conclusions

This study provides robust evidence of the relationship between comorbid PCMD and AD in men and PCMD in their wives via gender-based violence in a low-income Southeast Asian country. This suggests that the mental health and behaviours of men in this setting exert a powerful effect on the mental health of their wives during pregnancy and after childbirth and that it is essential that strategies to screen and treat or prevent PCMDs in women will be of limited success unless they also address comorbid PCMD and AD in men. The data also indicate that research to investigate these relationships using comparable techniques to those used in this study in low- and high-income settings are required.

## Abbreviations

AD: Alcohol dependence; PCMDs: Perinatal common mental disorders; SEM: Structural Equation Modeling.

## Competing interests

We declare that we have no conflicts of interest.

## Authors’ contributions

TDT, TT and JF participated in all phases of the study, including the original idea, design, data collection, data analysis and interpretation. KW contributed to analysis of data. All authors contributed to writing of the manuscript. All authors read and approved the final manuscript.

## Pre-publication history

The pre-publication history for this paper can be accessed here:

http://www.biomedcentral.com/1471-244X/12/148/prepub
